# Proteome-wide analysis of lysine 2-hydroxyisobutyrylation in *Frankliniella occidentalis*

**DOI:** 10.1186/s12864-022-08841-w

**Published:** 2022-08-29

**Authors:** Chengying Ding, Liyun Song, Ying Li, Lili Shen, Dongyang Liu, Fenglong Wang, Zhonglong Lin, Jinguang Yang

**Affiliations:** 1 Key Laboratory of Tobacco Pest Monitoring, Controlling and Integrated Management, Tobacco Research Institute of Chinese Academy of Agricultural Sciences, Qingdao, 266101 China; 2 Liangshan State Company of Sichuan Province Tobacco Company, Liangshan, 615000 China; 3 Country Yunnan Province Company of China Tobacco Corporation, Kunming, 650001 China

**Keywords:** *Frankliniella occidentalis*, Proteome, Post-translational modifications, Lysine 2-hydroxyisobutyrylation, Pathogenicity

## Abstract

**Background:**

Lysine 2-hydroxyisobutyrylation (Khib) is a novel and conserved post-translational modification (PTM). *Frankliniella occidentalis* are economically important agricultural pests globally and also notorious for vectoring destructive plant viruses. To better study the disease transmission mechanism of *F. occidentalis*, it is necessary to conduct in-depth analysis of it. So far, no Khib modification of insects has been reported.

**Results:**

In this study, a proteome-wide analysis of Khib modifications in *F. occidentalis* was analyzed for the first time through the combination of high performance liquid chromatography fractionation technology and 2-hydroxyisobutyrylated peptide enrichment and other advanced technologies, 4093 Khib sites were identified on 1125 modified proteins. Bioinformatics and functional enrichment analyses showed that Khib-modified proteins were significantly enriched in many cell compartments and pathways, especially related to various cellular components and biological processes, and were more concentrated in ribosomes and proteasome subunits, involved in energy metabolism, protein synthesis and degradation, compared to the other nine species including Japonica rice, *Homo sapiens*, *P. patens*, *Botrytis*, *Ustilaginoidea virens*, *Saccharomyces cerevisiae*, *T. gondii*, *C. albicans*, and *F. oxysporum*. And Khib sites on virus-interacting insect proteins were discovered for the first time, such as cyclophilin and endoCP-GN.

**Conclusions:**

After three repeated experiments, we found a total of 4093 Khib sites on 1125 proteins. These modified proteins are mainly concentrated in ribosomes and proteasome subunits, and are widely involved in a variety of critical biological activities and metabolic processes of *F. occidentalis*. In addition, for the first time, Khib modification sites are found on the proteome of *F. occidentalis*, and these sites could be acted as for the virus interaction, including cyclophilin and endoCP-GN. The global map of 2-hydroxyisobutyrylation in thrips is an invaluable resource to better understand the biological processes of thrips and provide new means for disease control and mitigation of pest damage to crops.

**Supplementary Information:**

The online version contains supplementary material available at 10.1186/s12864-022-08841-w.

## Introduction

 Post-translational modification (PTM) of protein is a critical link in the intricate mechanism of life. In PTMs, single or multiple modification groups are used to connect amino acid residues on post-translational proteins to modulate intramolecular and intermolecular interactions. PTMs also change the physical and chemical properties of the protein and affect its spatial conformation, active state, subcellular positioning, folding, stability, and interaction, ultimately affecting its function [1, 2]. All the way from protein synthesis to degradation, PTMs play a vital role [3, 4]. PTMs emerge considerably faster and more diverse than the synthesis of new proteins, additionally, they're also required for the production of new proteins. They can assist in the adaptation and survival of organisms in an ever-changing environment, as well as being a component of important biological pathways that govern a wide range of cellular physiological activities [5]. Lysine residues have been shown to include many novel forms of PTMs, including propionylation [6], crotonylation [7], succinylation [8], malonylation [9], glutarylation [10], 2-hydroxyisobutylation [3] and so on, collectively referred to as lysine acylations. These modifications can change protein function by regulating different cellular processes [11]. The roles of these modifications vary among species, but they are all crucial to life activities [12, 13], it is of great value to further explore the roles of these modifications in species.

Lysine 2-hydroxyisobutyrylation (Khib), an unique post-translational lysine acylation mechanism initially described in 2014, is a novel type of lysine acylation [14]. It is evolutionarily conserved with wide distribution in almost all creatures [14]. The structure of lysine 2-hydroxyisobutyrylation is unique from that of other modifications, such as acetylation. Notably, because of the presence of hydroxyl groups, lysines modified with Khib may establish hydrogen bonds with other molecules [14]. According to reports, numerous Khib are found in plants, such as *Oryza sativa*, *Triticum aestivum L.*, *Arabidopsis thaliana*, *Nicotiana tabacum*, and *Physcomitrella patens* [15–18]. Systematic proteomic lysine 2-hydroxyisobutylation studies on tobacco, *Arabidopsis*, rice, corn, and other plant species have revealed that Khib is a conservative form in plants and that this modification is involved in carbohydrate metabolism and plant stress adaptation [17]. Researchers have also found it is present in a variety of biological pathogens, such as in the animal pathogens *Proteus mirabilis*, *Toxoplasma gondii*, and *Candida albicans* [19–21]. Khib has been shown to be widespread in plant pathogens as well, such as *Fusarium oxysporum*, *Botrytis cinerea*, *Ustilaginoidea virens*, and *Aspergillus niger* [22–26]. Analysis of these four plant pathogens shows that Khib is broadly distributed among cell compartments and participates in various cellular processes, including the regulation of pathogenicity and virulence [22, 26, 27]. Khib also exists in animals, for example, H4K8 (H4 histone Lys8 site) 2-hydroxyisobutylation (H4K8hib) was detected in the active transcription gene of mouse meiosis and meiotic cells [14], and further experiments have shown that H4K8hib is involved in carbon stress-related functions and regulation [28]. Through histone analysis and the rapid regeneration of H4K8 protein after glucose starvation, H4K8 was clearly found to be dependent on the glycolytic pathway, which revealed the enrichment of this modification in glycolysis/gluconeogenesis in mice [28]. However, little is known about Khib in insects. Moreover, the effect of 2-hydroxyisobutylation on thrips is almost completely unknown, especially its function in thrips’ life activities and disease transmission.

Vector-borne viruses are an important worldwide agricultural problem. Most plant-infecting viruses are spread through arthropods [29, 30]. The most effective vectors are insects with piercing mouthparts, and these mouthparts are used to inject the virus into specific plant tissues [31]. It can cause immeasurable damage to high-value crops and ornamental plants [32]. There are four main types of thrips that are harmful: western flower thrips (*Frankliniella occidentalis* Pergande), onion thrips (*Thrips tabaci* Lindeman), melon thrips (*T. palmi* Karny), and yellow tea thrips (*Scirtothrips dorsalis* Hood) [32]. Thrips can spread many plant viruses, such as tomato blight virus (TBV), soybean vein necrosis virus (SVNV), tobacco streak virus (TSV), and impatiens necrotic spot virus (INSV) [33, 34]. Among them, tomato spotted wilt orthotospovirus (TSWV) is a circulating continuous propagation virus with the greatest host range, genome organization, at this stage, its most effective transmission vector is *F. occidentalis* [35]. Vectors offer a strong environment for viral replication to continue, and the midgut cells and principal salivary glands of *F. occidentalis* are two major tissues for TSWV replication [36, 37]. It can be seen that thrips are a very harmful medium of plant virus transmission. The control status of *F. occidentalis* was studied to find effective methods to control and reduce its harm to agriculture. At present, the use of a variety of pesticides to control this pest results in resistance to multiple pesticides and associated outbreaks of secondary pests and do not achieve the intended control purpose, as well cause major environmental issues [32]. We need to research new weapons to control thrips and other sucking plant pests. At this stage, only a few reports have described PTMs in insects [38], and none describe Khib in thrips. We performed a comprehensive proteome analysis of Khib in *F. occidentalis* and discovered 4093 Khib sites on 1125 proteins to investigate the mechanism of action of Khib in thrips and its influence on cell function. These discovered proteins were found in abundance in *F. occidentalis* cells and acted as a cofactor in a variety of biological processes. We analyzed the Khib site motifs as well as modified protein structures and function; compared the conservation of the discovered proteins with the modified proteins of other species; and summarized the pathogenic related proteins. This study is critical for elucidating the process of virus propagation through thrips, and its management.

## Material and methods

### Thrips culture

*F. occidentalis* used in the study were originally collected from clover plants, *Trifolium repens* L. (*Fabales: Fabaceae*), at the Experimental Station of Qingdao Agricultural University. These thrips were raised on lentils under a 16-h light/8-h dark photoperiod at 28 °C. A mixed sample of female and male thrips was taken and used immediately for protein extraction.

### Protein extraction and trypsin digestion

The thrips (0.3 g per biological reuse) were ground into powder using liquid nitrogen, and the powder was sonicated three times using a high-intensity ultrasonic processor (Scientz, Ningbo, China) in lysis buffer (8 M urea, 2 mM Ethylene Diamine Tetraacetic Acid (EDTA), 3 µM Trichostation (TSA), 50 mM nicotinamide (NAM), 10 mM Dithiothreitol (DTT), and 1% protease inhibitor cocktail) on ice. The remaining cellular debris was centrifuged at 21,000 g for 10 min at 4 °C, and the supernatant was kept. Finally, 15% Tri-Chloroacetic Acid (TCA) was added to the solution and incubated for 2 h at -20 °C to precipitate the protein. The mixed solution was centrifuged at 4 °C at 12000 g for 10 min to discard the supernatant. The precipitate was separated and acetone washed three times. The protein was redissolved in buffer (8 M urea and 100 mM NH_4_CO_3_, pH 8.0), and determine the final protein concentration using the 2-D Quant kit (GE Healthcare).

For digestion, the protein solution was alkylated with 11 mM iodoacetamide for 15 min at room temperature in darkness after being treated with 5 mM DTT for 30 min at 56 °C. Triethylamonium bicarbonate (TEAB) buffer (100 mM) was added to the protein solution to make sure urea conc becomes less than 2 M to remove the effects of urea on trypsin digestion. For the first step, trypsin was added at a 1:50 trypsin to protein mass ratio overnight, and at a 1:100 trypsin to protein mass ratio for the second 4-h digestion.

### Western blot assay

Proteins were extracted as described above, separated by 12% SDS-PAGE at 120 V and for 100 min, and then transferred to polyvinylidene fluoride (PVDF) membranes. After blocking with blocking solution (5% BSA, 20 ml 1X TBS-T) for 1 h, immunoblotting was performed using pan anti-Khib multiclonal antibody (Micrometer Biotech Company, Hangzhou, China).

### High-performance liquid chromatography fractionation

The peptides were fractionated through high-pH reversed-phase high-performance liquid chromatography (HPLC) using the Agilent 300 Extend (Agilent) C18 column [particles, 5 μm; inside diameter, 4.6 mm; and length, 250 mm]. HPLC is very suitable for separating protein mixtures in the initial stage. Peptides were separated into 80 fractions over an 80-min period using a gradient of 2%-60% acetonitrile (pH 10) in 10 mM ammonium bicarbonate. The peptides were then mixed into a single fraction and vacuum centrifuged to dry them.

### Affinity enrichment

To isolate and enrich Khib-modified peptides, we incubated HPLC purified tryptic peptides dissolved in NETN buffer (100 mM NaCl, 1 mM EDTA, 50 mM Tris–HCl, 0.5% NP-40, pH 8.0) with pre-washed agarose-conjugated anti Khib antibody beads at 4 °C overnight with gentle shaking (Micrometer Biotech, Hangzhou, China). The beads were subsequently washed with NETN buffer four times and then with ddH_2_O two times. The bound peptides were eluted from the beads with 0.1% trifluoroacetic acid. Finally, the eluted fractions were combined and vacuum dried. For LC–MS/MS analysis, the resulting peptides were desalted with C18 Zip Tips (Millipore) according to the manufacturer’s instructions.

### LC–MS/MS analysis

The affinity enriched tryptic peptides were diluted in 0.1 percent formic acid (solvent A) and loaded directly onto a reversed-phase column pre-constructed (Acclaim PepMap 100, Thermo Scientific, Waltham, MA, United States). Following that, the peptides were separated using a reversed-phase analytical column (Acclaim PepMap RSLC, Thermo Scientific). The gradient comprised an increase from 6 to 22% solvent B (0.1% formic acid in 98% acetonitrile) over 24 min, 22% to 40% in 8 min, increasing to 80% in 3 min, and finally 80% for the last 3 min, all at a constant flow rate of 400 nL/min using an EASY- nLC 1000 UPLC system.

The peptides were analyzed with an NSI source through tandem mass spectrometry (MS/MS) in Q Exactive TM plus (Thermo Scientific) and coupled with UPLC online. The electrospray voltage was 2.0 kV. The complete peptide was detected in Orbitrap at a resolution of 70,000. A peptide was selected for MS/MS and NCE was set to 30. Ion fragments were detected in the Orbitrap at a resolution of 17,500. A data-dependent procedure, alternating between 1 MS scan and 20 MS/MS scans, was used for the first 20 precursor ions with a threshold ion count of > 5E3 in the MS survey scan, and the dynamic elimination time was 15.0 s. Automatic gain control was used to prevent interference of the orbiter, and 5E4 ions were collected to generate MS/MS spectra. For the MS scans, the m/z scan range was 350–1,800. 100 m/z was chosen as the fixed initial mass. Micrometer Biotech Company performed the LC–MS/MS analysis (Hangzhou, China).

### Database search

The Maxquant search engine (v.1.5.2.8) was used to process the resultant MS/MS data. Tandem mass spectrometry spectra were compared to the SwissProt Thrips database and the reverse decoy database. Trypsin/P was specified as the cleavage enzyme that allowed 5 modifications per peptide, 5 charges, and as many as 4 deletion cleavage. The mass error was set to 10 ppm for precursor ions and 0.02 Da for fragment ions. Carbamidomethyl on Cys was specified as a fixed modification, and Khib modification, oxidation on Met, and 2-hydroxyisobutyrylation both of lysine residue and protein N-termini were specified as variable modifications. The threshold for proteins, peptides, and modification sites was 1% [24]. The minimum peptide length was 7. All of the other settings in MaxQuant were set to their defaults. The chance of choosing a place was set to more than 75%.

### Bioinformatics methods

The model of sequences containing amino acids in certain places of modified-21-mers in all protein sequences was analyzed using MoMoV5.0.2 [39]. All proteins found in databases were used as background databases. The minimum number of times the word was used was set at 20. The “emulate original motif-x” option was chosen, and the other settings were left as they were. Through statistical analysis of the amino acid sequence before and after all 2-hydroxyisobutylation sites in the sample, the regular trend of the amino acid sequence in the region where 2-hydroxyisobutyrylation occurs was calculated. Then, WOLFSPORT was used to predict, classify, and analyze the subcellular structure. WOLFSPORT is an updated version of PSORT/PSORT II for predicting eukaryotic sequences.

We adopted a bioinformatics analysis method that could organically link the various information of genes and proteins to provide the required information. The GO annotation proteome was derived from the UniProt-GOA database (www. http://www.ebi.ac.uk/GOA/) [40]. First, the identified protein IDs were converted to UniProt IDs, and then, they were mapped to GO IDs according to the protein IDs. If some identified proteins were not annotated by the UniProt-GOA database, InterProScan v.5.14–53.0 (http://www.ebi.ac.uk/interpro/) was used to predict the protein’s GO function based on the protein sequence alignment method [41]. Subsequently, GO annotation was used to retrieve the corresponding information from the protein database, and the khib-modified proteins were further classified on the basis of the three categories: biological process, cellular component, and molecular function [42].

The KEGG database, an information network linking known molecular interactions, was used to annotate protein pathways [43]. First, using a KEGG online service tool, KAAS v.2.0 (http://www.genome.jp/kaas-bin/kaas_main), the protein’s KEGG database description was annotated. The annotation result was then mapped to the KEGG pathway database using another KEGG online service tool, the KEGG mapper V2.5 (http://www.kegg.jp/kegg/mapper.html). The domain function description of the identified 2-hydroxyisobutylated protein was annotated by the InterPro domain database All the aforementioned bioinformatics analyses, that is, GO, KEGG pathway, and protein domain enrichment, were performed using the two-tailed Fisher’s precision test. A corrected *p* value of < 0.05 for each GO, KEGG pathway, and protein domain term was considered significant.

To determine the degree of evolutionary conservation of 2-hydroxyisobutylation, BLASTP was used to compare the sequence of the 2-hydroxyisobutylated protein in thrips with the specific 2-hydroxyisobutylated protein sequence. By applying the best BLAST attack method (sequence similarity > 30% and E value < e^−5^), we identified homologous proteins among these proteins. For each homology group, we used MUSCLE v3.8.31 for multiple sequence alignment. For all identified Khib proteins (related to amino acid metabolism), we searched the name identifier of protein–protein interaction (PPI) in the STRING database version 10.03 [44]. Only the interactions between proteins belonging to the search data set were selected, thereby excluding external candidate proteins. STRING uses an indicator termed confidence to define the confidence of the interaction; we selected all interactions with a confidence of 0.9 (high confidence). The interactive network from STRING was visualized in Cytoscape (Version 3.3.0). The molecular complexity detection graph of the theoretical clustering algorithm was used to analyze dense connected regions.

## Results

### Identification of 2-hydroxyisobutyrylated proteins in thrips

**Fig. 1:**
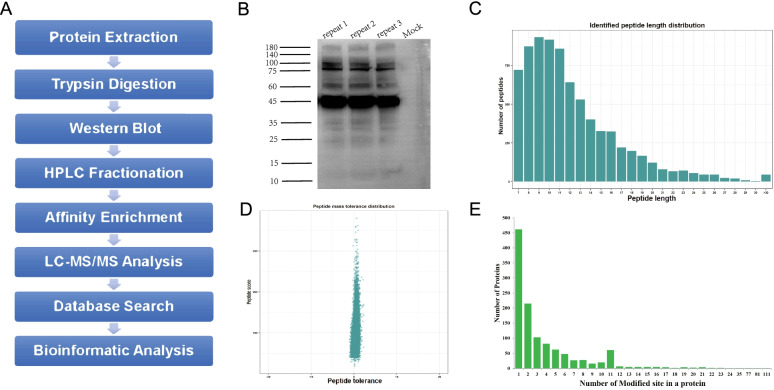
Identification of 2-hydroxyisobutyrylated proteins in thrips. **A** Overview of experimental procedures used in this study. **B** Immunoblot analysis with pan anti-Khib antibody in *F. occidentalis*, each protein lane represents a biological replicate (Figure S1). **C** Peptide length distribution. **D** Mass error distribution of all identified peptides. **E** Number of modified sites in a protein

In order to identify the Khib sites in the thrips, we combined affinity enrichment and proteomics technology to systematically analyze Khib (Fig. 1A). Under the same experimental conditions, three sets of experimental procedures were repeated to identify Khib proteins and sites in thrips. To further confirm the modification, the whole protein in *F. occidentalis* was immunoblotted using Khib pan-antibody, and immunoblot had three biological repetitions, with similar results. (Fig. 1B). Mass spectrometry was used to acquire a total of 48,686 secondary maps. Searching the maps and comparing them for protein theoretical data revealed that 28,717 maps could be effectively used, and the map utilization rate was 58.98%. Among the 7698 peptides identified by analyzing the map, 6997 (90.98%) had Khib modifications. In total, 1125 proteins were identified, which had 4093 Khib-modified sites (Table S1, S2). To verify our MS data, we performed quality control. The majority of peptides were 7–20 amino acids in length, which conformed to the general trypsin enzymatic hydrolysis and higher-energy collision dissociation (HCD) fragmentation rules and thus met the quality requirements of mass spectrometry samples (Fig. 1C). Due to the great precision nature of the Orbitrap mass spectrometer, the mass error of the majority of mass spectra was less than 10 ppm, suggesting that the mass spectrometer's mass accuracy was normal and that the protein's qualitative and quantitative analysis findings satisfied the standard (Fig. 1D). We computed the number of modification sites on each protein to identify the distribution of Khib sites. We discovered that 462 modified proteins included just one Khib site, but proteins containing two or more modification sites accounted for more than half of all modified proteins. The number of proteins containing 10 or more Khib-modified sites was as many as 134. It can be seen that this modification is widespread in thrips proteins (Fig. 1E). This data is available through ProteomeXchange and ID number is PXD030798. Sequencing results revealed 20,885 total proteins in thrips (*F. occidentalis*. fasta), and proteins with the 2-hydroxyisobutyl group accounted for 5.39% of the total proteins (1125/ 20,885). Therefore, we believe that Khib is a common and complex protein PTM in thrips.

### Analysis of Khib site motifs

**Fig. 2:**
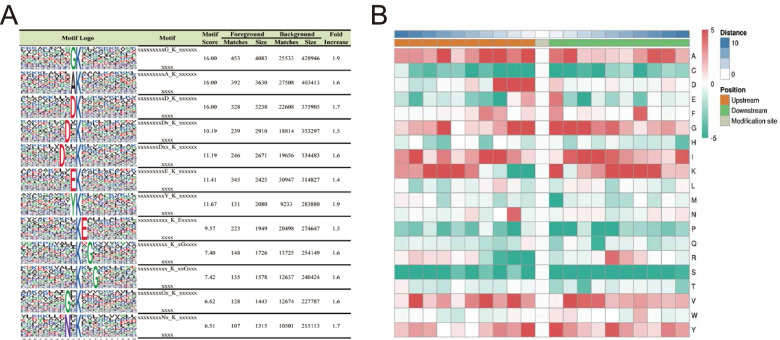
Analysis of the Khib site motifs. **A** Motif analysis shows Khib peptide motifs and conservation of Khib sites. The intensity map shows enrichment of amino acids in particular positions around Khib lysine residues. **B** Heat map of the amino acid compositions around Khib sites. Red indicates enrichment and green indicates depletion

To determine whether a common sequence motif was present in the Khib peptides, the MoMo software was used to compare the amino acid sequence around the Khib sites to all thrips background sequences (Table S3). Around the Khib site, we identified ten conserved motifs in the thrips (Fig. 2A). In particular, the motifs of xxxxxxxxxG_K_xxxxxxxxxx, xxxxxxxxxA_K_ xxxxxxxxxx, and xxxxxxxxxD _K_ xxxxxxxxxx (motif score > 15.00) were very conserved. Among the 7698 peptides, 10 amino acids were identified upstream and downstream of the Khib site (− 10 Khib + 10), accounting for 90.8% (6997/7698) of all identified peptides. EKhib, K(X5) Khib, DKhib, and AKhib (X is an unspecified amino acid residue) were also found in species such as *B. cinerea* [22], *F. oxysporum* [45], *Myzus persicae* [46], and *A. niger* [25], indicating that they are all conserved in Khib-modified motifs of differet species (Fig. 2B). According to heat map analysis, the amino acid frequencies flanking the Khib site were as follows: alanine acid (A) at positions − 5 to − 1, + 1, and + 2; aspartic acid (D) at positions − 1, − 2, and − 3; glutamic acid (E) at positions − 1 and + 1; glycine (G) at positions − 1, − 2, + 1, + 2, and + 3; lysine (K) at positions − 10 to − 5, + 1, and + 5 to + 10; and valine (V) at positions − 4 to − 2 and + 2 to + 4. Tyrosine (Y) at positions − 1, + 1, and + 5 facilitates 2-hydroxyisobutyrylation.

### Structural analysis of all 2-hydroxyisobutyrylated proteins

**Fig. 3:**
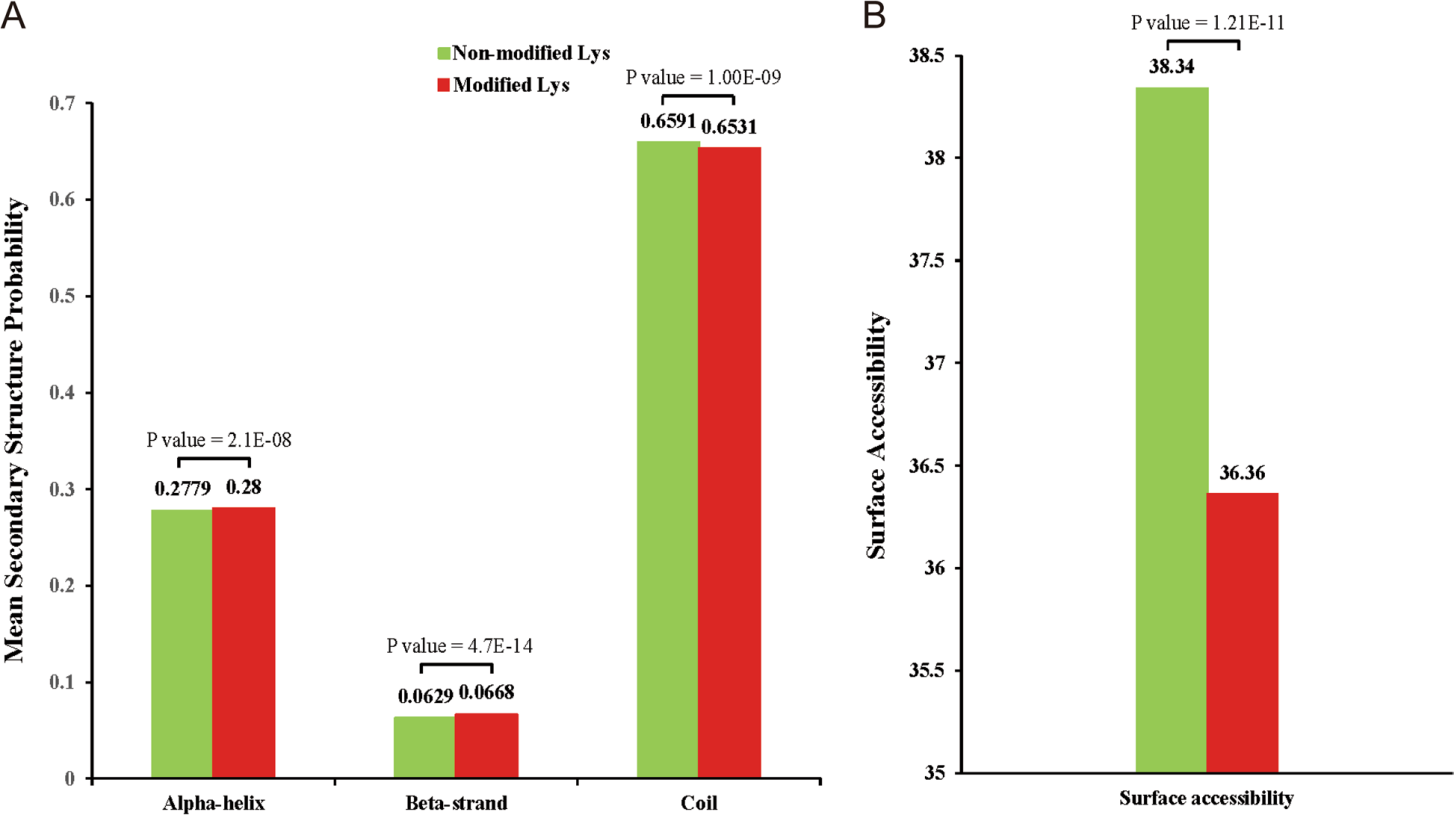
Structural analysis of all 2-hydroxyisobutyrylated proteins and predicted surface accessibility of Khib sites

NetSurfP was used to perform secondary structure analysis and detect the preferred structure of the Khib site in the protein. A total of 65.3% of the Khib sites were located in the coiled region, 28.0% of the sites were located in the alpha-helix, and 6.68% of the sites were located in the beta-strand. This distribution between 2-hydroxyisobutyrylated lysine and unmodified lysine is very similar (Fig. 3A). However, the 2-hydroxyisobutyrylated sites were less accessible on the surface than the unmodified regions (Fig. 3B). The surface accessibility was higher in non-modified sites and lower in Khib sites, therefore in way, the occurrence of Khib may have an impact on protein tertiary structure, which in turn might affects protein function and makes these proteins play an important role in biological processes.

### Conservation analysis of khib sites in different species

**Fig. 4:**
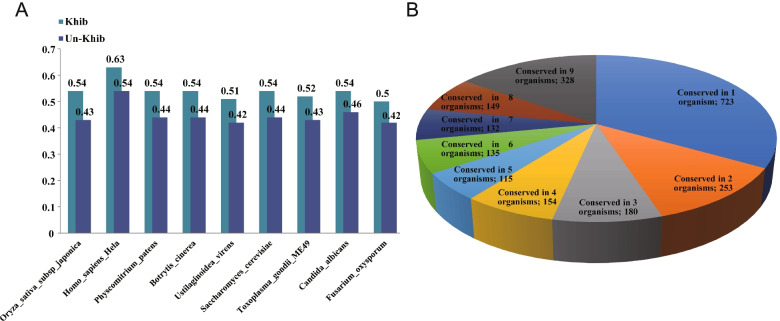
Test of the evolutionary conservation degree of Khib in different species. **A** Proportion of conserved Khib proteins of 9 distant species to total modified proteins. **B** A pie chart of conservation of Khib sites in 9 organisms

Firstly, we used BLASTP to compare the 2-hydroxyisobutyrylated protein sequences of western flower thrips with the specific Khib protein sequences of 9 distant species to test the degree of evolutionary conservation of Khib, such as Japonica rice, *Homo sapiens*, *P. patens*, *Botrytis*, *Ustilaginoidea virens*, *Saccharomyces cerevisiae*, *T. gondii*, *C. albicans*, and *F. oxysporum* (Table S4). Through the best BLAST calculation method, we found 8838 Khib sites in the nine species in the protein databases. The proportion of Khib-modified conserved lysines of Japonica rice, *H. sapiens*, *P. patens*, *Botrytis*, *U. virens*, *S. cerevisiae*, *T. gondii*, *C. albicans*, and *F. oxysporum* was 54%, 63%, 54%, 54%, 51%, 54%, 52%, 54%, and 50% (Fig. 4A), further parallel comparison of thrips with nine other species at the same time showed that 328 highly conserved Khib sites were found together in ten species, and the proteins containing these modified sites accounted for 11.5% (129/1125) of the total protein number of thrips. There are about 723 unique Khib sites in different species. These data suggest that different species-specific Khib sites may have special functions, and that conserved sites are of great significance to life activities in different species (Fig. 4B and Table S4).

### Functional annotation and cellular localization of Khib proteins in thrips

**Fig. 5:**
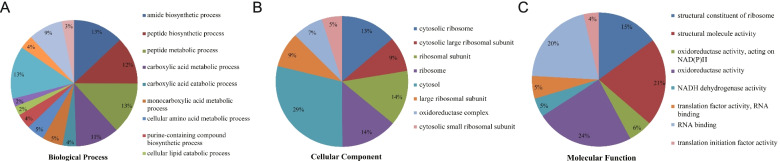
Pie charts showing the distribution of Khib proteins. **A** Khib proteins categorized according to the biological process. **B** Khib proteins categorized according to the cellular component. **C** Khib proteins categorized according to the molecular function

On the basis of GO item classification analysis, we classified the activities of thrips Khib proteins into biological processes, cellular components, and molecular functions for our investigation (Table S5). Among biological processes, the largest number of proteins (147, 271, and 282 proteins) were involved in amide biosynthesis, carboxylic acid catabolism and metabolism, and peptide biosynthesis and metabolism, respectively. These proteins accounted for 62% (1125) of the identified modified proteins (Fig. 5A). Among cellular components, the cytoplasm and organelles were the most numerous places for Khib protein, especially ribosomes, account for approximately half of the total identified proteins. Additionally, the Khib protein was discovered to be dispersed throughout the cellular matrix. (Fig. 5B). In terms of molecular function, the majority Khib proteins were involved in oxidoreductase activity (270), structural molecule activity (236), and NADH dehydrogenase activity (225), and as a structural constituent of ribosomes (169) (Fig. 5C). Analysis of the subcellular localization of Khib proteins showed that the identified proteins were mainly located in the cytoplasm (425 proteins), nucleus (194), cytoskeleton (169), and endoplasmic reticulum (138) of thrips, accounting for approximately 12%-38% of the total identified proteins (Table S6). The little number of proteins discovered in the extracellular matrix, nucleus, mitochondria, plasma membrane, and other areas accounted for around 2%-7% of the total identified proteins. These findings indicated that Khib proteins are widely distributed in thrips, especially in important cellular structures, may be involved in their various biological functions.

### Function, pathway, and domain enrichment analyses of Khib proteins in thrips

**Fig. 6:**
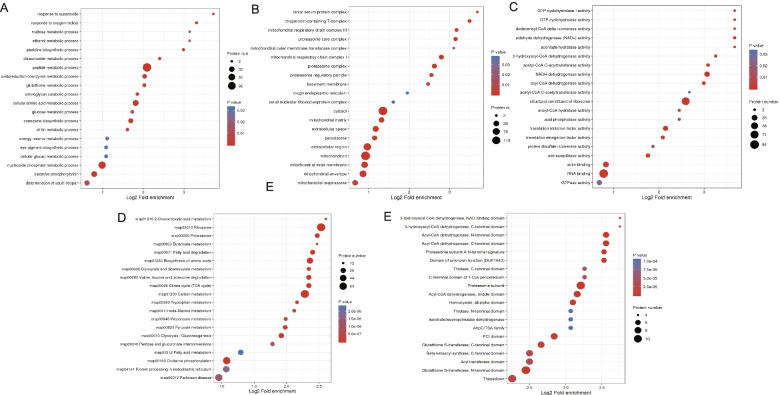
Enrichment analysis of 2-hydroxyisobutyrylated proteins in thrips. **A**, **B**, **C** GO enrichment analysis was based on the biological process, cellular component, and molecular function. **D** KEGG pathway-based enrichment analysis *(*www.kegg.jp* kegg / kegg1.html)*. **E** Protein domain enrichment analysis of all identified proteins

We conducted GO enrichment, KEGG pathway, COG/KOG category, and protein domain studies to further elucidate the putative roles of Khib proteins in thrips life activities. GO enrichment showed that (Table S7), among the cellular component category (Fig. 6A), a large number of proteins were enriched in the cytoplasm, ribosomes, and mitochondria. According to molecular function classification (Fig. 6B), these proteins may be involved in ribosome structure formation, RNA binding, oxidoreductase activity, GTP hydrolase activity, translation initiation factor, elongation factor activity, and peroxidase activity. Proteins involved in biological processes, such as peptide metabolism, redox coenzyme metabolism, cellular amino acid metabolism, nucleoside phosphate metabolism, and oxidative phosphorylation, may have undergone 2-hydroxyisobutylation modification (Fig. 6C).

We conducted further investigations of the Khib proteins of thrips using KEGG pathway and protein domain analysis (Fig. 6D and Table S8). Khib proteins were highly abundant in 20 thrips pathways according to the KEGG pathway enrichment study. Among all the 20 enrichment pathways, the two most enriched pathways were related to ribosomes (map03010) and carbon metabolism (map01200). The results are consistent with the GO analysis results. These two pathways were related to protein synthesis and productivity. In addition, we identified another 18 enrichment pathways, including those related to intracellular protein synthesis and processing (e.g., amino acid biosynthesis (map01230), glyoxylic acid metabolism, and dicarboxylic acid metabolism (map00630)). Other pathways were mostly related to energy production, such as carbon metabolism (map01200), oxidative phosphorylation (map00190), citric acid cycle (TCA cycle) (map00020), glycolysis/gluconeogenesis (map00010), pyruvate metabolism (map00620), propionic acid metabolism (map00640), butyric acid metabolism (map00650), and fatty acid degradation (map00071).

Numerous findings from the protein domain enrichment study corroborated the results from the GO and KEGG databases. The identified Khib proteins were enriched in 20 domain families (Fig. 6E and Table S9). The most enriched protein domains were proteasome subunits, proteasome subunit-N-terminal feature, acyl-CoA dehydrogenase-N-terminal domain, acyl-CoA dehydrogenase-C-terminal domain, acyl-CoA dehydrogenase-middle domain, 3-hydroxyacyl-CoA dehydrogenase-N-terminal domain, glutathione S-transferase- N-terminal domain, and PCI domain. Functional enrichment analysis further refines the distribution of Khib and revealed that Khib proteins were not only widely distributed but also played a key role in cell synthesis and metabolism in thrips.

### Khib proteins are involved in central metabolism

Khib modification was detected in a substantial number of proteins involved in the ribosome and proteasome, as well as proteins related to energy metabolism, as determined by the KEGG pathway enrichment study. Among them, more than half of the large subunit unit and small subunit unit proteins of the ribosome were modified by Khib, especially numerous modification sites were observed in the initiation factor, elongation factor, and ribosome translocation channel complex (Fig. S2A). The three domains of the proteasome complex showed various modified proteins (Fig. S2B), particularly the standard product except α5, β6, and β7. The remaining 11 proteasome subunits were all modified and belonged to the core particle (20S proteasome). In addition, Khib-modified proteases are involved in every step of the metabolic pathways, such as glycolysis pathway, TCA cycle, fatty acid metabolism in mitochondria, and oxidative phosphorylation. The results are consistent with those of GO enrichment and protein domain enrichment analyses. Compared with other life activities, Khib modification is more concentrated in protein synthesis and degradation, as well as energy metabolism.

### PPI network of Khib proteins in thrips

**Fig. 7:**
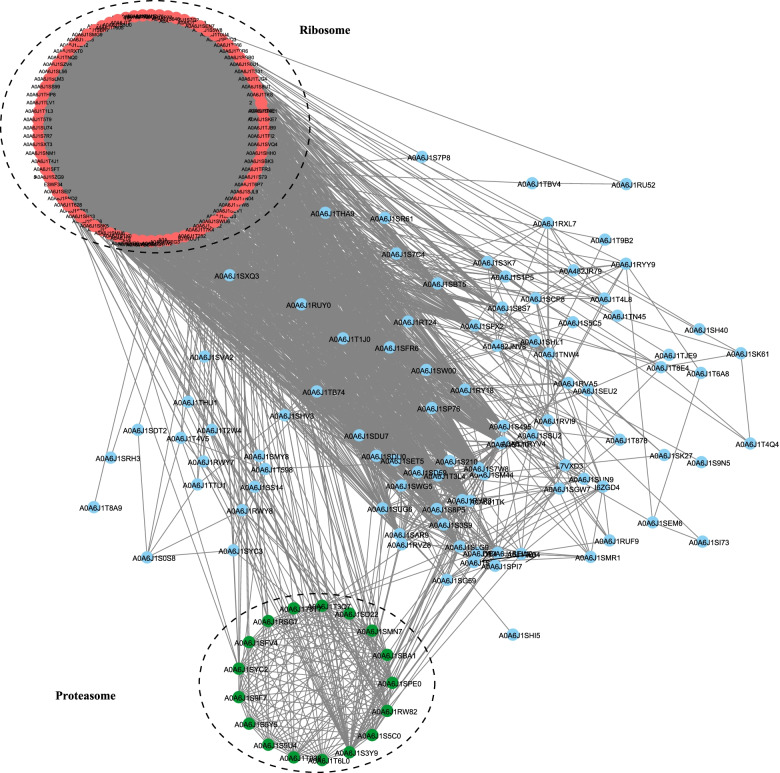
The PPI network of Khib proteins in thrips. Two highly interconnected Khib protein clusters are related to the ribosome and proteasome. These clusters were represented by different colors and black dots

To determine the correlation between 2-hydroxyisobutyrylated proteins in *F. occidentalis*, we generated a PPI network of Khib regulatory proteins of thrips based on the STRING database. Numerous modified proteins linked together to form a highly interconnected protein network. Subsequently, molecular complexity detection (MCODE) analysis showed that all the 2-hydroxyisobutyrylated proteins were tightly connected. It also revealed that Khib proteins were closely related to various protein modification processes and metabolic pathways in the life activities of *F. occidentalis*. Numerous complexity and cellular functions organized together into conspicuous and densely linked clusters, especially those related to ribosomes and proteasomes, enriched with numerous modified proteins (Fig. 7 and Table S10). These results are consistent with those of GO enrichment, KEGG pathway enrichment, and other analyses, indicating that the identified proteins regulated multiple pathways of *F. occidentalis*. These data were helpful for further analyzing the function of dihydroxybutyryl modification and infer its vital role in protein synthesis, degradation, and metabolism, highlighting the value of Khib in crucial biological processes of thrips.

### Analysis of Khib proteins involved in pathogenesis in thrips

**Fig. 8:**
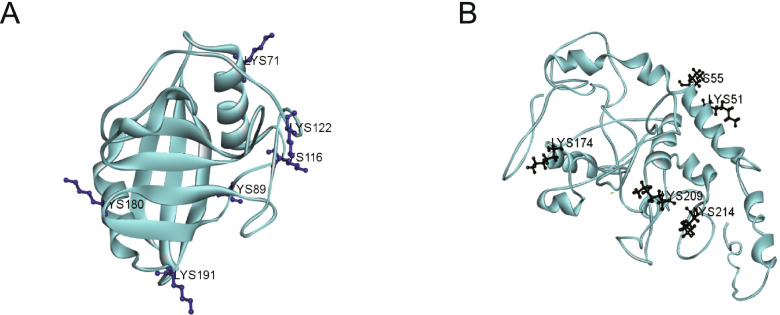
Three-dimensional structure of two thrip proteins involved in pathogenesis with the identified Khib site. **A** Cyclophilin. **B** endoCP-GN. The structure was predicted by I-TASSER. The recognized sites are highlighted in black

*F. occidentalis* is an important virus transmission vector and is of significance for studying the proteins that interact with the virus. Many proteins of *F. occidentalis* that are related to the transmission of TSWV and other viruses were found to undergo 2-hydroxyisobutylation modification. The midgut and salivary glands of *F. occidentalis* are the most important tissues in the virus reproduction process and can stably express viruses such as TSWV, one of the most representative proteins is a type of the cuticular proteins (CP proteins) [33, 36, 47], endoCP-GN (GenBank accession: MH884757), had 5 Khib sites (K52, K55, K174, K209, and K214) (Fig. 8A and Table S1). Another modified protein, called cyclophilin (GenBank accession: MH884760), had 6 Khib sites (K71, K89, K116, K122, K180, and K191) in *F. occidentalis* (Fig. 8B and Table S1). This protein was not only involved in metabolic activities but was also closely related to the TSWV–GN interaction [36]. Cyclophilin is proven to promote or prevent viral infections [48, 49]. It has also been shown to have a key function in the transmission of grain yellow dwarf virus using aphids as a carrier [50]. The above results show to some extent that Khib modification may played a regulatory role in the sustainable transmission of virus through western flower thrips.

## Discussion

Proteomics is still an essential tool for studying insect morphological development and pest management at this stage. Recent research in insect proteomics shows that multiple modifications are associated with disease transmission and host defense mechanisms [50–53]. PTMs are involved in the conversion of normally inactive precursor proteins to mature proteins, and they affect the structure and function of a wide variety of proteins [54], Numerous protein modifications are required for the morphological development and disease transmission processes of insects [46, 55]. PTM is a key means to promote protein functional diversity, and studying PTM is of great significance. Khib, a newly discovered PTM, has been reported in mammals, plant, yeast, bacteria, and fungi [15, 17, 20, 25, 28, 56], but no exploratory research has been done on insects.

As an important vector for transmitting viral diseases to commercial crops [57], proteome study of thrips may provide a novel method for preventing and controlling disease spread. In this study, Khib protein analysis was conducted using the whole proteome. In total, 4093 Khib sites were identified on the 1125 modified proteins in *F. occidentalis*, accounting for approximately 5.4% of the proteome, it was proved that Khib was widespread in *F. occidentalis*. Using the enrichment analyses of GO and KEGG pathways and cluster of Orthologous Groups of protein (COG/KOG) (Fig. S3) mutual mapping analysis, 2-Hydroxyisobutyrylated proteins were found to be present in many metabolic cycles and interconnected with the biological activities involved, for example, a large number of modified proteins were distributed in ribosomes and proteasomes, and were involved in glycolysis, gluconeogenesis, oxidative phosphorylation, fatty acid degradation pathways, and TCA cycle, these metabolic pathways were closely related to the activities of RNA processing and modification, energy production and conversion, protein synthesis and fatty acid elongation degradation. These analytical results suggested that Khib may play a significant role in *F. occidentalis*’s fundamental life processes.

Amino acid motif analysis of proteins’ modified showed that Khib in *F. occidentalis* has a substrate preference (selectivity), amino acids such as negatively charged aspartic acid exhibit strong bias around the positions of Khib, they are similar to that of other species (Japonica rice, *H. sapiens*, *P. patens*, *Botrytis*, *U. virens*, *S. cerevisiae*, *T. gondii*, *C. albicans*, and *F. oxysporum*), and different kinds of amino acids have a certain deviation near the modified position of Khib, these findings indicated that the amino acid sequence position of lysine has a major influence in Khib modification. Through the study of 328 Khib sites identified as fully conserved or highly conserved across 10 species found that these Khib sites are present in 129 proteins of *F. occidentalis* that have similar functions in 9 other species, including the structural components of ribosomes, translation, initiation, mRNA and rRNA binding, protein folding, energy and protein metabolism, and other metabolic pathways, and are mostly distributed in the cytoplasm and ribosomes. A total of 723 unique Khib sites were found on different proteins in different species, each species contains unique Khib modified proteins and participates in the specific life activities of the species, for example, this modified protein is involved in the biosynthesis of antibiotics in *Candida albicans* [21], and in photosynthesis in rice [56]. Compared with other species, this modification was more concentrated in the ribosomes and proteasomes of *F. occidentalis*, this finding was more directional for the studies on modification function of it. These data suggest that Khib is widely present in different species, and may have basic biological functions, and may have specific functions due to different species.

An insect is an indispensable factor in the spread of disease. It transmits viruses from infected plants to healthy hosts through various transmission strategies [58]. Vector proteins play an important role in the reproduction and infection of viruses [59], such as Cyclophilin and endoCP-GN can promote virus reproduction and transmission in many insect vectors [31, 36, 60], for example, *F. occidentalis* is an important TSWV transmission vector, and its cyclophilin, endoCP-GN and other proteins can interact with viruses to promote disease transmission [31], the Whitefly Bemisia tabaci Cyclophilin B can interact with tomato yellow leaf curl virus (TYLCV) in the whitefly midgut to promote virus circulation and transmission [60], the CP of *Laodelphax striatellus* interacts with rice stripe virus’s nucleocapsid protein (pc3) [61], and the CP of *Rhopalosiphum padi* interacts with the read-through protein of the barley yellow dwarf virus (BYDV) [51], the study of this type of protein is particularly important. By digging into the omics information, for the first time, we had discovered Khib sites on thrips’ virus-interacting proteins, such as cyclophilin, and endoCP-GN, all have dihydroxyisobutyryl modifications at multiple sites. By analyzing the research results of the pathogenic mechanism of dihydroxyisobutyrylation in other species, we found that Khib plays a non-negligible role in disease occurrence, for example, Khib positively regulates the disease resistance of rice infected with *Ustilaginoidea virens* [62], and can promote the virulence factor of *Ustilaginoidea virens* [23]. We speculate that Khib plays a role in disease development. These findings provide new ideas for the study of the mechanism of arbovirus transmission. The in-depth study of Khib-modified protein of *F. occidentalis* is of great significance for controlling the spread of the disease.

In short, the extensiveness within the individual and conservation between species reflect the value of lysine 2-hydroxyisobutyrylation studies. We found a total of 4093 sites on 1125 proteins after three repeated trials, giving a rich data set for Khib in insects. Bioinformatics analysis showed that these modified proteins are involved in a variety of critical biological activities and metabolic processes of thrips, may also be implicated in host infection and reaction to unfavorable environmental variables. The global map of 2-hydroxyisobutyrylated in thrips is a valuable resource for better comprehension of the biological processes of thrips that allow them to spread disease and harm the crops. The Khib modification of the virus-interacting protein in thrips may be a key factor in the vector’s ability to transmit viruses and therefore can be targeted for disrupting the virus disease cycle in insect vectors. 

## Supplementary Information

**Additional file 1:** 

## Data Availability

The mass spectrometry proteomics data have been deposited to the ProteomeXchange Consortium via the PRIDE partner repository with the dataset identifier PXD030798 (http://www.ebi.ac.uk/pride/archive/).

## Abbreviations

*F. occidentalis*: *Frankliniella occidentalis*
Khib: Lysine 2-hydroxyisobutyrylation
PTM: Post-translational modification
H4K8hib: H4K8 2-hydroxyisobutylation
HPLC: High-pH reversed-phase high-performance liquid chromatography
KEGG: Kyoto Encyclopedia of Genes and Genomes
GO: Gene Ontology
COG/KOG: Cluster of Orthologous Groups of protein
MCODE: Molecular complexity detection
TSWV: Tomato spotted wilt orthotospovirus
TYLCV: Tomato yellow leaf curl virus
BYDV: Barley yellow dwarf virus
TBV: Blight virus
SVNV: Soybean vein necrosis virus
TSV: Tobacco streak virus
INSV: Impatiens necrotic spot virus
BSA: Bovine Serum Albumin
PVDF: Polyvinylidene fluoride
TSA: Trichostation
NAM: Nicotinamide
DTT: Dithiothreitol
EDTA: Ethylene Diamine Tetraacetic Acid
TCA: Tri-Chloroacetic Acid
TEAB: Triethylamonium bicarbonate
